# Selective internal radiation with Y-90 resin microspheres (SIRT) for liver metastases of gastro-intestinal stromal tumors (GIST) resistant to tyrosine kinase inhibitor (TKI) therapy

**DOI:** 10.1038/s41416-025-02952-3

**Published:** 2025-03-05

**Authors:** Peter Hohenberger, Nils Rathmann, Karen Büsing, Franka Menge, Jens Jakob, Daniel Pink, Eva Wardelmann, Stefan O. Schoenberg, Steffen J. Diehl

**Affiliations:** 1 Division of Surgical Oncology and Thoracic Surgery, University Medical Center Mannheim, Medical Faculty Mannheim – Heidelberg University, Heidelberg, Germany; 2 Institute of Clinical Radiology and Nuclear Medicine, University Medical Center Mannheim, Medical Faculty Mannheim – Heidelberg University, Heidelberg, Germany; 3 Department of Surgery Mannheim University Medical Center, Medical Faculty Mannheim, Heidelberg University, Mannheim, Germany; 4 Sarkomzentrum Berlin-Brandenburg, HELIOS Klinikum Berlin-Buch, Berlin, Germany; 5 Gerhard Domagk Institute of Pathology, University Hospital Muenster, Muenster, Germany

**Keywords:** Sarcoma, Sarcoma

## Abstract

**Background:**

Hepatic metastases of GIST might be the dominant site of progression and resistant to available tyrosine kinase inhibitors (TKIs). Selective internal radiation therapy (SIRT) offers treatment by intratumoral radiation up to 200 Gy. We analyzed the hepatic progression-free survival (H-PFS) in a consecutive patient cohort.

**Methods:**

Twenty-six patients (median age 57.6 years) with biopsy proven liver metastases of GIST were treated by SIRT. All had RECIST documented tumor progression, and 24/26 patients had up to four lines of pretreatment. Mutational status was ‘quadruple wildtype’ (q-wt, *n* = 5), KIT exon 11/9/13 in *n* = 15/4/1 cases and PDGFRα (*n* = 1). Median follow-up of this retrospective analysis of a prospectively kept database is 33.6 months.

**Results:**

Median H-PFS was 16 months (range, 4–54+ months, 95% CI 6.5–25.4 months) and OS after SIRT was 28 months (95% CI 17.2–28.7 months). Best H-PFS was observed in patients with ‘q-wt’ at 25 months (range, 6+–54 months, 95% CI 16.2–33.8 months). The worst outcome was for KIT exon 11 mutations plus secondary mutations with 7 months (range, 4–33 months, 95% CI, 4.2–9.8 months).

**Conclusions:**

90Y-SIRT is a potent treatment for patients with liver metastases of GIST resistant to TKI therapy. In patients with ‘q-wt’ GIST, SIRT is an option for first-line use.

## Introduction

Gastrointestinal stromal tumors (GIST) are the most common subtype of soft tissue sarcomas [1] originating predominantly in the stomach and small bowel [2]. R0 resection is the mainstay of treatment for locally confined primary tumors. There is a well-evaluated option of downsizing locally advanced primary tumors by neoadjuvant treatment with imatinib in sensitive tumors harboring mutations in *KIT* exon 11 [3].

The liver and peritoneum are the predominant areas of metastatic spread in GIST. Tumor dissemination almost always is multifocal and thus systemic treatment is indicated. Distant metastases can be controlled by therapy with registered drugs of imatinib [4], sunitinib [5], regorafenib [6], and ripretinib [7] contributing to long-term disease control for patients with sensitive mutations in the *KIT* protooncogene [8–10]. In patients with metastatic GIST and mutations in the *PDGFRα* gene, avapritinib is a registered treatment option [11]. The decisive element for successful drug therapy is the tumor-driving mutation, f.e. *KIT*, *Braf* [12] *or NTRK* [13]. For patients suffering from hepatic metastases of GIST only [14], liver resection is not recommended by ESMO guidelines [15] or NCCN mainly due to the fact that continuation of antiproliferative treatment is required even after surgical removal [15, 16].

Two cohorts of patients do not or no longer profit from systemic treatment. The one group consists of patients with GIST not harboring a drug-sensitive mutation, so-called quadruple wildtype tumor (q-wt, i.e. no *KIT*, *PDGFRα*, BRAF or SDH mutation [17–19]). The other group of patients suffer from the development of 2^nd^/3^rd^-ary mutations while being treated with 1^st^/2^nd^/3^rd^/4^th^ line tyrosine kinase inhibitors (TKI). The acquired resistance mutations result in the fact that metastases do no longer respond to drug therapy [9, 20, 21]. Once secondary mutations or non-tolerable toxic side effects of drugs limit systemic therapy, local treatment of progressive metastases might be an option. Patients fulfilling the criteria of oligometastatic disease [22, 23] are the most adequate candidates then. Thermal ablation by microwave or radiofrequency as well as chemoembolisation or cryoablation [24] play a role in early hepatocellular cancer and all methods were also explored in GIST [25–27].

If multiple hepatic metastases are the only or major area of tumor progression and are resistant to available drug treatment with TKIs as described above, radio-embolization with yttrium-90 (^90^Y) microspheres is a promising tool, applying local radiation doses up to 200 Gy. Selective internal radiation therapy (SIRT, synonymous transarterial radioembolization TARE) is an accepted method for treating colorectal liver metastases and recommended by guidelines from European Society of Medical Oncology (ESMO) or National Comprehensive Cancer Network (NCCN) in case of chemotherapy-refractory situations [15, 28]. A systematic review suggested that the treatment could be cost-effective as a monotherapy as well as combined with systemic chemotherapy [29].

We indicated this approach for a selected group of patients suffering from biopsy-proven metastatic GIST with either no further drug option after all available TKIs had been administered or with no option for drug treatment due to q-wt status. Our initial experience on 11 patients was published earlier [30]. Here, we analyze the long-term results of SIRT with respect to hepatic progression-free survival (H-PFS) as the primary endpoint in a consecutive cohort of 26 patients and evaluate patient subgroups with respect to their mutational status.

## Patients and methods

### Patients

This is a single-center analysis of a consecutive patient cohort with unresectable liver metastases of GIST with prospective follow-up and treated by one therapeutic team. From February 2008 to March 2022, 26 patients (14f, 12m, median age 51 years, range, 17–72 years) with histologically confirmed GIST with biopsy proven hepatic metastases underwent SIRT with ^90^Y-SIR resin microspheres (Sirtex Co., Woburn, MA, USA). Clinical data of the patients are listed in Table 1. The majority of patients were recruited from our large outpatient facility as a tertiary referral center for sarcoma patients, five patients had been treated elsewhere and were referred specifically for SIRT but had all follow-up examinations at our center.

**Table 1:** Clinical data of the patients treated.

Number of patients	*n* = 26
Males/females	14/12
Age (median, range) at GIST diagnosis	51.8 (17–72) years
Age (median, range) when receiving SIRT	57.6 (18–75) years
ECOG	
Score 0	*n* = 20
Score 1	*n* = 6
Primary tumor location	
Stomach	*n* = 8
Duodenum	*n* = 2
Small bowel	*n* = 14
Rectum	*n* = 2
Patients with primary metastatic disease disease (M1)	*n* = 12
Median time to M1 disease (*n* = 14)	29.3 months (range, 6–55 months)
Type of mutation	
*KIT* exon 11 mutated patients	*n* = 8
*KIT* exon 11+ secondary mutation	*n* = 7
*KIT* exon 9	*n* = 4
*KIT* exon 13	*n* = 1
*PDGFRα* D842V	*n* = 1
Quadruple wt (q-wt) patients	*n* = 5
Time from M1 disease onset to SIRT	Median 35.5 months (range, 4–128 months)
Number of treatment lines prior to SIRT and	
In *KIT* mutated patients (*n* = 21)	Median 3, (range, 1–5)
In q-wt patients (*n* = 5)^a^	Range 0–4, two patients without prior drug

^a^Treatment lines in 3 patients (1): sorafenib stopped for toxicity (2), imatinib 400 mg and imatinib 600 mg + everolimus (3), imatinib 400 mg to 800 mg, sunitinib, nilotinib, imatinib 600 mg + everolimus.

*Indication for treatment*: The patients’ hepatic disease was not amenable to surgical removal of liver metastases with clear margins and radiofrequency ablation (RFA) could not reach all progressive lesions in the liver due to anatomical or technical limitations. Additionally, extrahepatic disease if present had to be controlled by antiproliferative TKI at the time of decision-making for SIRT. All patients had an ECOG score of 0 (*n* = 20) or ECOG 1 (*n* = 6) and histologically proven liver metastases of GIST which were the only (*n* = 16) or the clearly dominant site of progression (*n* = 10). All patients showed RECISTv1.1 tumor progression and were presented to a multidisciplinary sarcoma board which gave the recommendation to proceed with SIRT. In patients with tumor progression at hepatic and extrahepatic sits no SIRT was indicated. Three patients with extrahepatic disease continued their drug treatment during SIRT therapy.

In all but two patients, available standard TKIs had been shown to be unable to control hepatic tumor progression and no further drug treatments were available at the given time or thought to be indicated due to the mutational status. The median number of treatment lines prior to SIRT was three and varied dependent on availability of TKIs over time and access to trials with new drugs. Drug treatment included imatinib and sunitinib in the overwhelming majority of patients and regorafenib, pazopanib, masitinib, nilotinib, sorafenib and everolimus, with several of the drugs being administered within prospective clinical trials. In more recent years and with the documented efficacy of SIRT [30], we indicated first line therapy in two females with q-wt tumor or Carney-Stratakis syndrome.

### SIRT treatment

All patients fulfilled the criteria to indicate SIRT recently published [28]. The guidelines on criteria to the use of liver‐directed therapies for nonsurgical management of liver metastases by the American Radium Society [31] clearly were not known during the study period. However, the individual indications for our patients with SIRT would have been supported by the guidelines at the given time.

According to our standard operating practice (SOP) [30], all patients received a pretherapeutic work-up by contrast-enhanced CT and MR-tomography to locate vessels variants. To assess to hepato-pulmonary shunt volume, a conventional scintigraphy was performed. Via a transarterial approach, ^99m^Tc–labeled macroaggregated albumin (MAA) was injected to both liver arteries (typically 60 MBq to the left and 90 MBq to the right liver artery) and distribution of the radionuclide was measured over the lungs and the liver an set in relationship. Shunt volumes below 10% from liver to lung were accepted prior to SIRT. For shunt volumes between 10 and 20%, the radioembolisation dose had to be adapted. Two patients with shunt volume exceeding 20% had to be excluded from the procedure. Only patients with levels of bilirubin <2 mg/dl, SGOT/GPT < 4 f UL and INR > 50% underwent radioembolization.

To prevent embolization to nontargeted radio-sensitive organs several arteries (right gastric, gastroduodenal, cystic and other small-caliber arteries) were occluded using platinum microcoils in the same session of the MAA study or before administering Y-90 SIR spheres. As a premedication to SIRT, patients received 60 mg of prednisolone, 1000 mg of paracetamol, 7.5 mg of piritramid and 8 mg of ondansetron as part of our SOP.

Twenty-two patients received treatment of both liver lobes with a mean dose of 1,71 GBq (range, 0.96–2.5 GBq) Y-90 SIR spheres. In four patients only one liver lobe was treated with a range of 0.95–1.88 GBq (Supplementary Table 1). All patients received just one dose of Y-90 SIR spheres. Dose calculations used the body surface area (BSA) method, which was the only method accepted by the company to use their spheres when we started first treatments [32]. After SIRT, laboratory changes were closely monitored and classified according to CTCAE criteria to rule out radiation-induced liver dysfunction (RILD) or liver failure.

### Response assessment

Initial therapy response was assessed by MRI performed four months after SIRT, consisting of contrast-enhanced MRI (CE-MRI) and diffusion-weighted imaging (DWI) including so-called apparent diffusion coefficient (ADC) maps. The scans were classified as either regression (partial or complete), stable disease or progression of the predetermined and SIRT targeted liver tumors according to Choi [33], taking into account the change of the contrast-to-noise ratio. For progression assessment afterwards, multicenter follow-up MRI and CT scans were used, initially every 3 months. The modality and frequency at later times depended sometimes on the clinical condition of the patients. The progression classification was based on the final assessments of the corresponding radiologist using the combined criteria by Choi [30, 34, 35].

### Follow-up

All patients were followed-up after SIRT at our institution until death, median follow-up time after SIRT is 25 months (range, 4–147 months, 95% CI 16.8–33.1 months). Laboratory of values were recorded during follow-up and the most significant deviation from normal range within 3 months after SIRT was used to classify toxicity according to Common Terminology Criteria for Adverse Events (CTCAE) [36].

### Statistical analysis

Descriptive data, survival calculations and graphical representation were performed with IBM-SPSS version 27. Descriptive data are given as median, range and with 95% confidence intervals. We calculated the time interval from the date of conducting SIRT to the date when progression was documented. Of each patient, overall survival (OS), post-SIRT survival (pSS), and hepatic progression-free survival (H-PFS) were estimated by using the Kaplan–Meier method and the comparison of H-PFS between independent groups was performed by using the log-rank test. OS was defined as the period from the initial diagnosis of the primary tumor to the date of death and pSS from the date of SIRT to the date of death. The cause of death was not considered for survival calculation but is depicted together with details of the clinical course and the underlying molecular findings of the mutational status in Table 2. The H-PFS was defined as the period from the SIRT to the first progression at the location of the Y-90 SIR spheres application. In case of progression at extrahepatic sites (eH-PFS) was calculated separately.

**Table 2:** Description of the clinical course and individual treatment of SIRT patients.

Gender	Age	Time to M1	M1 to SIRT	#lines pre	SIRT resp.	H-PFS	1stPD post SIRT	#lines post	OS post SIRT	Status	Mutational type
f	22	55	50	3	CR	21	HEP at 21 mos	3	87	DOD	Ex 11 N556_573del + **D820Y**
f	43	10	96	3	SD	33	OSS at 6 mos	2	33	DOD	Ex 11 M552-V559del + **Y823D**
m	39	44	78	4	PR	8	HEP at 8 mos	2	21	DOD	Ex 11 W557_K558del + **Y823C**
m	43	Initial M1	128	5	SD	4	HEP at 4 mos	3	13	DOD	Ex 11 31bpdel + **D820Y**
m	55	36	7	2	PR	15	HEP at 15 mos	3	26	DOD	Ex 11 w557_558del + **V654A**
m	53	Initial M1	37	2	PR	6	HEP at 6 mos	2	12	DOD	Ex 11 K558_E562del + **N822K**
f	39	Initial M1	93	4	PR	5	PER at 3 mos	2	7	DOD	Ex 11 W557_558delins + **V654A**
f	69	41	39	3	SD	4	PER at 3 mos	0	4	DOD	Ex 11 W557R
m	68	24	34	3	SD	34	OSS at 20 mos	3	36	DOD	Ex 11 L576P
f	72	Initial M1	20	2	CR	6	HEP + PER at 6mos	1	28	DOD	Ex 11 W557_559del
m	70	Initial M1	31	3	SD	4	HEP at 4 mos.	2	12	DOD	Ex 11 W557_V559del
m	49	Initial M1	72	2	PR	15	HEP at 15 mos	1	21	DOC^#^	Ex 11 W557_558Kdel
f	67	45	13	3	CR	29	OSS at 25 mos	4	78	AWD	Ex 11 dupl 1724_1765
f	63	17	107	1	SD*	22	PER at 8 mos	3	23	DOD	Ex 11 K558_V559del
m	65	Initial M1	109	3	SD	26	OSS at 3 mos	3	34	DOD	Ex 11 K550_K558del
f	49	10	24	4	PR	13	PER + STS at 6 mos	2	19	DOD	Ex 9 Y502_503F ins
m	48	Initial M1	74	3	PR	7	HEP + PER at 7mos	2	16	DOD	Ex 9 Y502_503F ins
m	42	48	13	1	PR	30	HEP at 30 mos	2	147	AWD	Ex 9 Y502_503F ins
f	42	24	66	4	SD	5	OSS + STS at 4mos	0	10	DOD	Ex 9 Y502_503F ins
m	65	Initial M1	67	4	PR	7	HEP at 7 mos	2	12	DOD	Ex 13 K642E
f	46	6	40	1	PR	16	PER at 5 mos	2	66	AWD	PDGFRA Ex 18 D842V
m	68	39	17	4	PR*	25	PER at 15 mos.	1	40	DOD	qwt
f	46	12	33	1	PR	32	PER at 11 mos	5	77	AWD	qwt
f	36	Initial M1	10	2	PR	23	PER at 3 mos	4	32	DOD	qwt
f	17	Initial M1	5	0	SD	41		0	54	AWD	Carney Stratakis syndrome
f	57	Initial M1	5	0	SD	6		0	6	AWD	SDHB-deficiency
					*pCR at biopsy					#post-operative lung embolism	

Gender: *f* female, *m* male, *Age* at disease onset in years, *Time to M1* time interval after treatment of the primary tumor until the detection of metastatic disease in months, *M1 to SIRT* duration of metastatic disease from detection to treatment with SIRT in months, *#lines pre* number of treatment lines prior to SIRT, *SIRT resp.* result of SIRT according to MRI-imaging. In two patients marked with*, a histological complete remission (pCR) was confirmed after biopsy, *H-PFS* duration of hepatic progression-free survival (H-PFS) after SIRT in months, *1stPD post SIRT* site of first progression after SIRT (*HEP* hepatic, *PER* peritoneal, *OSS* osseous, *STS* soft tissue), *#lines post* number of treatment lines after SIRT, *OS post SIRT* overall survival after SIRT (OS) in months, *Status* current status of the patients in months, *DOC*^*#*^ identifies death from lung embolism, (*AWD* alive with disease, *DOD* death of disease, *DOC* death of other cause), *Mutational type* mutational status of GIST, the mutation subtype in bold indicates the secondary mutation detected (*q-wt* quadruple wildtype).

## Results

**Fig. 1:**
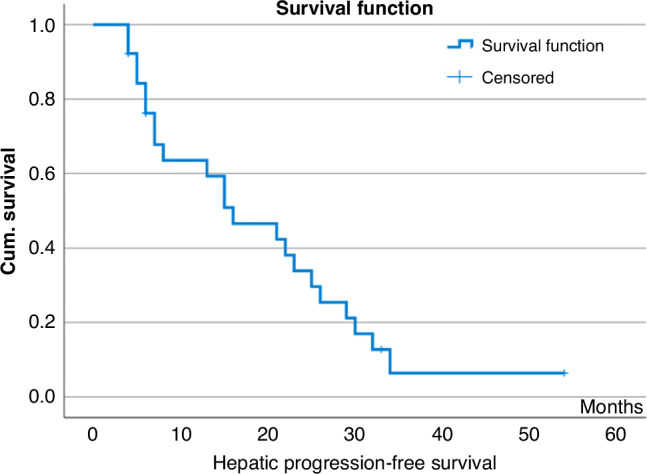
Kaplan–Meier estimates of hepatic progression-free survival after SIRT.

The median hepatic-progression free survival (H-PFS) after SIRT was 16 months (range, 4–54+ months, 95% CI 6.5–25.4 months), Fig. 1.

**Fig. 2:**
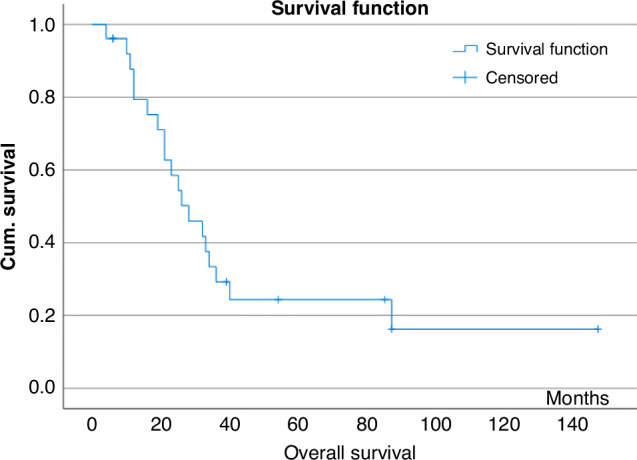
Kaplan–Meier estimates of overall survival of the total patient cohort after SIRT calculated from date of SIRT to death.

The median OS after SIRT was 28 months (95% CI, 17.2–28.7 months), six patients are alive at 6–78 months, Fig. 2.

Overall survival after diagnosis of the primary tumor was 102.5 months (range, 10–223 months, 95% CI 72.5–132.5 months), Table 2.

The median progression-free interval for the patients with extrahepatic disease was 6 months (range, 3–25 months, 95% CI 4.7–7.3 months).

There was no significant correlation between the time to metastatic disease after diagnosis of the primary tumor and post-SIRT survival.

### Influence of mutational status on H-PFS

**Fig. 3:**
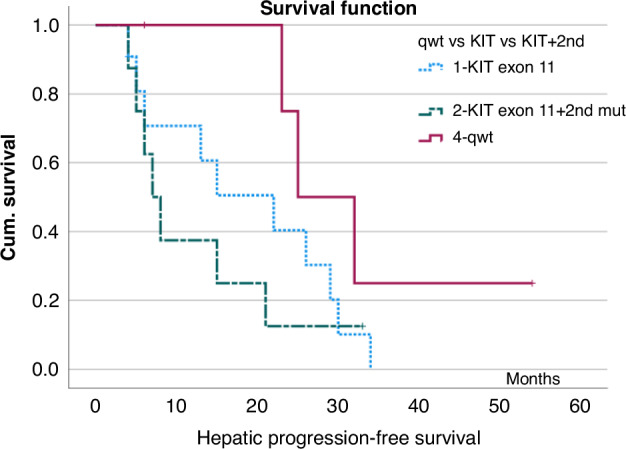
Kaplan–Meier estimates of hepatic progression-free survival (H-PFS) after SIRT, dependent on the mutational status, *p* = 0.067 log rank.

Median H-PFS in patients with a ‘quadruple-wildtype’ status (*n* = 5) was 25 months (range 6+–54 months, 95% CI 16.2–33.8 months). For patients with *KIT* exon 11 mutations without detectable secondary mutations median H-PFS was 22 months (range, 4–34 months, 95% CI, 8.4–35.6 months) whereas patients with known secondary mutations showed a H-PFS of only 7 months (range 4–33 months, 95% CI, 4.2–9.8 months, *p* = 0.16, log-rank), Fig. 3. When comparing the q-wt group (*n* = 5) with the total *KIT*-mutated group irrespective of secondary mutations (*n* = 19), the comparison was statistically not significantly different (*p* = 0.073, log rank). The overall group of patients with *KIT* exon 9/11/13 mutations showed a median H-PFS of 15 mos. (95% CI 5.2–24.8 months, *p* = 0.068 log-rank).

### Site of tumor progression

**Fig. 4:**
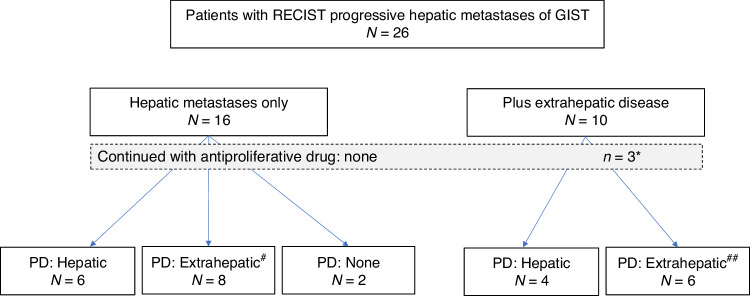
Site of tumor progression in relationship to the presence of extrahepatic metastases at the time of SIRT and continued systemic drug treatment. *indicates the drugs used after SIRT: sunitinib (*n* = 2, for two and 4 months), sorafenib *n* = 1 for 3 months. ^##^describes the location of progressive extrahepatic disease.

Initial site of tumor progression was the liver in 11 patients, in two of them in combination progression at peritoneal sites. Another seven patients developed peritoneal progression alone, while another two patients suffered from simultaneous progression at peritoneal and soft tissue sites (Table 2). Figure 4 splits patient groups with or without extrahepatic disease beyond liver progression. It can be shown that the control of liver metastases by SIRT was not co-induced by concomitant systemic treatment, Fig. 4. In the ‘hepatic only’ group 8/16 patients developed extrahepatic disease after SIRT. In the patient group suffering from additional extrahepatic disease, 6/10 patients progressed at extrahepatic sites. In total, only 10/26 patients with progressive liver metastases had primary progression in the liver, whereas 16 patients were not affected this way. Interestingly, in five patients bone metastases were the first site of progression after SIRT, see Table 2. Overall, seven patients developed bone metastases with a mean time interval of 17.1 months after SIRT (range, 3–43 months).

### Complications of treatment

Three patents suffered from major complications by developing gastric ulcers at 6, 7, and 17 months after SIRT requiring surgical resection. In one of them surgery had to be done as an emergency for perforation (CTCAE grade 4) and both other underwent elective segmental gastric resection for chronic ulcer (CTCAE grade 3). One patient was under sunitinib and another one under sorafenib therapy for extrahepatic tumor progression. The resection specimen showed resin microspheres at the basis of the ulcer. Another patient developed cholangitis that could be managed conservatively (CTCAE grade 2).

### Laboratory changes

Within a time range of 3 months after SIRT, changes in blood levels of hepatic enzymes showed CTCAE grade 3 in only one patient for bilirubin, who also showed grade 2 toxicity for ASAT and GGT. Five patients developed grade 2 toxicity, in four patients for GGT and in another one for bilirubin. Sixteen patients experienced grade 1 toxicity, typically as an elevation of ASAT (*n* = 14), ALAT (*n* = 12), AP (*n* = 12), GGT (*n* = 9), bilirubin (*n* = 12) and creatinine (*n* = 9) often in combination. No patient showed toxicity for INR comparing pre-SIRT versus post-SIRT values. Six patients (23.1%) did not show any CTCAE toxicity, (Supplementary Table 2).

### Post-SIRT treatment for tumor progression

After SIRT, patients received in median another three lines of TKI therapy for tumor progression (Table 2). Except of three patients, all other participated in at least one clinical trial for metastatic disease. Drugs administered and not yet mentioned above included avapritinib, cabozantinib, crenolanib, dasatinib, ponatinib, and ripretinib (in alphabetical order). Patients who developed bone metastases were treated with radiation therapy in parallel to TKI treatment.

## Discussion

Administration of locoregional radiation therapy using Y-90 has a history dating back to the 1950s in the setting of inoperable hepatocellular cancer (HCC) accessible by an intraarterial approach [37, 38]. Ariel et al. in 1950 used ceramic or glass microspheres with Y-90 sometimes in combination with intraarterial chemotherapy [39]. The method was not much in use after the 1980s with radiofrequency or microwave ablation (RFA) offering a less sophisticated and less invasive approach, however the power of the pure high-energy β-emitter was judged to be more promising than transarterial chemoembolization (TACE) for several tumor types in the decade from 1999 to 2009 [39].

For soft tissue sarcomas (STS), only scarce data on SIRT were published. A recent study reported on 39 patients with primary (*n* = 2) or metastatic liver sarcoma (*n* = 37) treated from 2006 to 2015. GISTs were excluded from the study due to their response to imatinib and in 14 different subtypes of STS the objective response rate was 36% [40]. Testa et al. treated two patients with liver metastases of GIST amongst 35 STS patients with liver-dominating progression resulting in a median OS of 20 months (95% CI: 13.9–26.1 months), while median H-PFS was 9 months (95% CI: 6.2–11.8 months). The objective response rate was 56.7%, and the disease control rate assessed by mRECIST at 3 months was 80.0%. No details were mentioned about the GIST patients and different response assessment criteria were used which complicates a comparison of treatment results [41].

To our knowledge, this series the largest reporting on the use of SIRT in GIST patients. The 26 patients represent a highly selected group accounting for only 2.45% of our 1060 GIST patients treated since 2004. We started the approach a decade ago due to the fact that tumor progression of liver metastases during treatment with TKIs was most often detected by increased contrast uptake [42, 43]. The hypervascularity also described as “nodule in a mass” [44] can be detected much earlier than tumor progression meeting RECIST [45–47]. Vital iodine tumor burden (VTB, [48]) is another component of tumor attenuation as a surrogate for enhancement or cell viability. We thought this to be the perfect rationale for a microsphere-based local transarterial treatment in patients with tumor progression being out options of drug therapy. In the majority of patients, we could document that polyclonal secondary mutations in the *KIT* gene were responsible for progression. This was initially detected from hypervascularized progressive metastases in comparison to avascular, still-responding other tumor deposits [20, 21]. After documenting success in the first 11 patients [30], we expanded the strategy also to patients known to have no active first-line drug treatment option due to the mutational status of their tumor. This refers particularly to GIST with SDH deficiency and those patients with a reference-pathology confirmed q-wt status. In our series those patients profited best from SIRT with H-PFI of 23, 25, 35 and 41 months respectively (Table 2). The subgroup shows the best treatment results and we therefore would advocate to administer SIRT as first line therapy in such candidate patients. SIRT therapy was successful in offering several patients a break in TKI treatment. Particularly, in those patients with liver metastases only (Fig. 4) there was a median of 9 months (range, 6–55 months) until proceeding to next line therapy. The drug-free interval is different from H-PFS, as availability of a next line and patient preferences (wish for pregnancy, fear of new side effects) played a major role.

The laboratory toxicity of SIRT in our patients was mild which is most probably to be attributed to the previous use of TKIs. In colorectal cancer patients, pretreatment with f.e. oxaliplatinum most often has already resulted in significant hepatotoxicity prior to SIRT. In the prospective CIRT study, 9% of the patients undergoing SIRT for different diseases experienced grade 3 toxicity [49] in comparison to just one patient in our series.

Gastric ulcer formation after SIRT is a long-known complication of treatment [50, 51]. Our rate of stomach complications requiring surgery (*n* = 3) is higher than in the CIRT study. All ulcerations were noticed after SIRT with a delay delayed by 6–17 months and all developed in the first nine patients, potentially as part of the radiologist’s learning curve. Twenty-four of 26 patients affected participated in clinical trials with meticulous clinical evaluation according to study plan. This work-up of side effects might be a bit more intensive in comparison to the CIRT register data [50]. Surgery was an emergency for perforation in one patient whereas both other patients showed a penetrating ulceration, one of whom being under sunitinib and another one under sorafenib treatment for peritoneal metastases. Resin microspheres could be detected within the ulceration and obviously the chronic inflammation in conjunction with TKI therapy known to hamper would healing had led to the complication. All patients had their gastric GIST primary tumor surgically removed prior to systemic treatment resulting in anatomical and vascular changes of the blood supply to the remnant of the stomach. In the CIRT study many patients had liver resection before SIRT, typically including a Pringle’s maneuver that might have occlude hepato-fugal blood flow by scar formation. A systematic review evaluated 29 publications on 51 patients with gastric ulcer after SIRT. Surgery had to be performed in seven patients, of whom only one patient had been treated for GIST liver metastases of GIST and very few of the patients had undergone stomach resection prior to SIRT [52]. Patients having had a gastric primary GIST removed and those being under TKI treatment for extrahepatic disease look to be at an increased risk for stomach ulcer formation.

Generally, treatment options for sarcomas metastatic to the liver are limited and local therapy is warranted as long as extrahepatic disease does not play a major role or is controlled [53]. This represents the inclusion criteria for SIRT in our patients. As a comparator of results of SIRT, TACE and RFA could function. Stereotactic radiation therapy (SBRT) has only been scarcely applied for liver metastases of GIST [54] and has not at all been used in a recent series of 113 patients fulfilling the criteria by ESTRO/EORTC to administer local therapy for oligometastatic disease [55].

RFA might be an alternative to SIRT as long as only a limited number of lesions needs to be treated and their location allows safe administration. In a series dating back to 2002, 29 patients with metastatic GIST were treated with ultrasound-guided RFA for primary or secondary tumor progression during drug therapy with imatinib [25]. The average number of target lesions was 2.3 (range, 1–8) and in 69 of 86 lesions, RFA was successfully performed. Thirteen patients had to be excluded from the procedure due to poor visibility. With a median follow-up of 33 months, six percent of the patients had local recurrence after 3.2–10.5 months. A retrospective series of 13 patients with GIST hepatic metastases reports that in 12 of 13 patients RFA was successful on the first approach with no data on H-PFS or additional treatment available [26]. A study on 100 patients including all subtypes of sarcoma (14 patients with GIST), evaluated the impact of liver ablation on the time interval free from systemic chemotherapy after ablation [27]. The patients were treated from 2007 to 2018 and half of them had failed previous systemic therapy. Nothing is reported about the location of metastases treated, thus the impact on controlling liver metastases remains speculative despite a reported chemotherapy-free interval of 14.7 months with the median not reached in the GIST patients. The authors concluded, that this treatment might improve patients’ quality of life without affecting their overall survival.

Regarding TACE, a reported describes 110 GIST patients treated from 1993 to 2005 with 12% of the patients developing PR followed by a median PFS of 8.2 months and a median OS of 17.2 months. Many of the patients received multiple TACE sessions [56]. In another series on 45 patients with liver-directed treatment of metastatic sarcoma treated from 2008 to 2013, six patients with GIST were reported, three of them treated as an emergency for tumor rupture and with a median OS of 76.8 months (95% CI, 32–94.1 months) [57]. Cao et al. compared embolization alone with TACE in the care of 45 patients with GIST and found better survival with embolization alone. Median OS was 74.0 weeks for embolization (95% CI: 68.2–79.8) versus 61.7 weeks for TACE (95% CI: 56.2–67.2 weeks) and the PFS in the TACE group was 42.1. weeks [58]. A most recent study reported the results of 238 patients with liver metastases of GIST treated with imatinib from 2002 to 2022 and analyzed the contribution of different adjunctive modalities hepatic resection (HR), RFA or TACE with 15% of the patients also suffering from extrahepatic metastases [59]. The authors compared the subgroups of imatinib alone (*n* = 126), imatinib + HR (*n* = 81) and imatinib + RFA/TACE(*n* = 31). The 10-year OS rate in the IM + HR group was significantly superior to the IM group and the IM + RFA/TACE group (91.9% vs. 61.1% vs. 55.2%, respectively. Obviously, some selection bias towards single liver metastases amenable to surgical removal may be assumed. In comparison to the results discussed, our median hepatic-progression free survival (H-PFS) after SIRT of 16 months with a 95% CI of 6.5–25.4 months clearly describes SIRT as the most promising treatment option for multifocal progressive liver metastases.

A nationwide survey on treatment of metastatic GIST from The Netherlands reported that 28% of the patients with liver metastases underwent local treatment in addition to systemic antiproliferative treatment with 12.2% of the patients having RFA whereas 15.8% underwent surgical removal of metastases [60]. In colorectal cancer metastatic to the liver it was discussed to use SIRT as a bridge to surgical resection [61]. To our knowledge no patient with GIST has undergone liver resection after SIRT. In our cohort, six patients with progression of liver metastases after SIRT were treated with different local measures (RFA, ablation with microwave, irreversible electroporation (IRE), or TACE). Future therapeutic options could use molecular imaging receptor targets like gastrin-releasing peptide (GRPR) known to characterize GIST cells and target it by radiopharmaceuticals [62].

In conclusion, our study documents that SIRT is a very valuable treatment in GIST patients suffering from progressive liver metastasis which cannot or no longer be controlled by TKI therapy offering a median of 16 months of hepatic progression-free survival. The approach is not a prefinal attempt in an otherwise desperate treatment situation. For this selected subgroup of patients treated for failure of all registered TKIs, the median hepatic PFS was 16 months, clearly better than PFS for 2nd or 3rd line drugs sunitinib (5) or regorafenib (6) with around 6 months and 4th line ripretinib with 8 months (7). Interestingly, during the long survival since disease onset seven patients seven developed bone metastases which otherwise is a rare event in GIST [63]. We do not think that there is any SIRT-specific mechanism for developing bone metastases. Our patients have had all available TKI treatment, were closely monitored and lived long enough to detect more sites of tumor dissemination than typical liver and peritoneum. Deducting indications for SIRT treatment in GIST patients, the best result can be expected in patients without known activating mutations in *KIT* or *PDGFR*α with a median of 25 months of H-PFS not offered by any other treatment option. At present, SIRT provides the most promising option for patients with SDH deficiencies, Carney-Stratakis syndrome, or Carney triad and could be advocated as first line therapy. Results from patients with *KIT*-mutated GIST are less encouraging and in patients with active extrahepatic metastases SIRT should be indicated very selectively.

## Supplementary information

Supplemental Table 1

Supplemental Table 1

Legends to supplemental tables

## Data Availability

The data that support the findings of this study are available on request from the corresponding author, [P.H.].
