# Supplementary material for: Selective internal radiation with Y-90 resin microspheres (SIRT) for liver metastases of gastro-intestinal stromal tumors (GIST) resistant to tyrosine kinase inhibitor (TKI) therapy
Source: Br J Cancer. 2025 Mar 5;132(8):716–24. doi: 10.1038/s41416-025-02952-3 (PMC11997030; doi:10.1038/s41416-025-02952-3)
Supplement: Supplementary file 3 — Legends to supplemental tables [file 41416_2025_2952_MOESM3_ESM.docx]

**Legends to supplemental tables**

Supplemental table 1

Dose of Y-90 SIR spheres administered in each single patient. RLL means right liver lobe, LLL means left liver lobe, in four patients only one lobe was treated.

Supplemental table 2
Details of laboratory toxicity of liver enzymes (ASAT, ALAT, AP, GGT, bilirubin), INR and creatinine and classification according to CTCAE after SIRT. Worst values measured within 3 months after SIRT were recorded.
